# Vulnerability to Ideologically-Motivated Violence Among Individuals With Autism Spectrum Disorder

**DOI:** 10.3389/fpsyt.2022.873121

**Published:** 2022-03-25

**Authors:** Marc R. Woodbury-Smith, Rachel Loftin, Alexander Westphal, Fred R. Volkmar

**Affiliations:** ^1^Biosciences Institute, Newcastle University, Newcastle upon Tyne, United Kingdom; ^2^Department of Psychiatry and Behavioral Sciences, Northwestern University, Feinberg School of Medicine, Chicago, IL, United States; ^3^Division of Law and Psychiatry, Department of Psychiatry and Child Study Center, Yale University, New Haven, CT, United States; ^4^Child Study Center, Yale University and Southern Connecticut State University, New Haven, CT, United States

**Keywords:** autism spectrum disorder, extremism, terrorism, vulnerability, mental health, incels, forensic

## Abstract

People with autism spectrum disorder (ASD) are represented among those who espouse extremist thought and have committed violent acts associated with their beliefs. Media often highlight a perpetrator's psychiatric diagnosis following acts of mass violence, which in some instances has included ASD. ASD may itself not provide useful information for understanding motivations. Instead, understanding specific traits and neuropsychological and other vulnerabilities may offer an opportunity to make sense of these very complex events.

## Introduction

Through the media we are often made aware of the mental health of individuals who have engaged in high profile acts of either mass violence or inexplicable acts of violence toward others (1, 2). Mental disorders such as schizophrenia and mood disorders are often cited as increasing the risk of unlawful behavior, although it is also recognized that this increased risk is modest (3, 4). More recent studies have also investigated mental disorders among people who have engaged in violence in the context of holding extremist beliefs. These indicate that among violent extremists between 10 and 17% have one or more mental disorders, with a range of psychiatric diagnoses represented, with mood disorder and schizophrenia the most frequent (5, 6). In recent years, the media has drawn attention to perpetrators who have been formally diagnosed with autism spectrum disorder (ASD) (2). Examples include the Toronto Van Attack and the Sandy Hook shootings, where, in both instances, the perpetrator had an established diagnosis of ASD. Similarly, among those who are drawn to extremist ideologies or outgroup affiliations, such as *incels* (involuntary celibates) (7), we are aware that a significant minority either have ASD or self-identify with this label (8). The perpetrator of the Toronto Van Attack is one such individual. Other examples do exist in the literature in which an ASD diagnosis is suggested (9, 10), but such highly-speculative cases will not be discussed further, particularly given the lack of formal diagnosis and other inherent biases in the approach taken (11).

It is important to understand the nature of any apparent relationship between diagnosis and behavior in such instances given that it may reflect aspects of vulnerability that characterize ASD and that may be amenable to intervention. Moreover, given that ASD as currently conceptualized has evolved into a relatively prevalent condition [more than 1:54 people, and with a year-on-year rise in prevalence of nearly 10%, (12)] it is increasingly important to understand the nature of this risk, including its implication for forensic mental health services and the criminal justice system. We set out to critically examine the evidence base concerning the relationship between ASD and acts of violence that are mediated by extremist beliefs. The relationship is, of course, complex, but, as discussed subsequently, red flags can be identified and, as such, it is crucial for mental health and criminal justice professionals to be alert to the possibility of ASD and its potential relevance.

## ASD as a Diagnostic Entity

ASD is an early onset, lifelong disorder impacting the core domains of social interaction and communication, and, by definition, is associated with concomitant functional impairment. ASD's social and communication vulnerabilities manifest in different ways and include difficulties such as initiating and maintaining social interaction with others, understanding non-literal aspects of communication such as irony and sarcasm, and cognitive difficulties in domains such as face and emotion processing, and planning, predicting, and problem solving. While poor problem solving and emotional regulation may predispose an individual to behave in a violent manner, a lack of empathy and interpersonal connection with others may increase the possibility that violent thoughts are enacted if there is a failure to conceptualize others as people who become victims. Much has been written about such mechanisms in the context of psychopathy (13). At a clinical level, of course, there are significant differences between ASD and psychopathy, but at a cognitive level there is some evidence of overlap (14, 15), so similar vulnerabilities may be relevant.

A number of other related symptoms also occur under the umbrella of rigidity of thought and behavior. These comprise strongly held beliefs and “black and white” thinking. Such beliefs can sometimes be abnormal or inappropriate in focus, and it is in this context that they may be deemed “extremist” in nature. Extremist thought may also appeal to those with a tendency toward black and white thinking because it can present a clear means of organizing an overwhelming and chaotic world. Belief that people can be readily classified by gender, race, or other features may make the world feel more manageable to someone who struggles to understand or follow social conventions. (It is worth noting that in the vast majority of people with ASD this tendency may have a very prosocial impact by encouraging rule-following.)

ASD has evolved into a broadly defined disorder whose margin relative to the “tail end” of population-level social and communication skills is somewhat blurred. Perhaps this distinction between those who formally meet diagnostic criteria and those who show traits but not the full clinical picture is not important, but that being the case, definitions will need to be adjusted accordingly. The prevalence of formally diagnosed ASD has seen an almost 10% year-on-year increase over the last 20 or so years (12). This increase is largely explained by a significant rise in diagnosis among those who function in the normal range of intelligence (i.e., an IQ of 70–75 or above) (12). Beyond the broadening of criteria, factors such as better recognition of the diagnosis and greater awareness among families and healthcare, social care, and education, explain this increase to a large extent. However, this is surely an under-estimate of actual prevalence, given that there will be other individuals who are unable to pursue a diagnostic assessment for health insurance reasons, and others who may live far from diagnostic availability. The data also support the fact that certain ethnic groups are under-represented (16). Additionally, those who do not identify with the illness model will never feature in healthcare statistics. This is important as there may be some individuals whose diagnosis does not become apparent until the occurrence of some event, such as alleged unlawful behavior.

## ASD and Unlawful Behavior

A number of previous studies have identified the risk of violence and other unlawful behavior among some people with ASD, and have expounded on some of the possible mechanisms (17). This is particularly true among the single case studies and case series that offer detailed investigation of relevant risk factors. There are a number of themes that seem to emerge consistently from this research, including a naïve understanding of relationships and jealousy of others or anger and frustration at their own experiences; being coerced into unlawful behavior by dominant others; and the obsessional pursuit of a particular interest (18). Other studies have also suggested that people with ASD are over-represented in secure psychiatric hospitals (19–21) and in prisons (22). Although such studies have suggested that as many as 5% of these populations are diagnosed with ASD, they are by no means robust in their methodology.

## ASD and Extremism

Extremism is characterized by beliefs that are held with conviction and with inherent hate and anger toward others. As such, those holding such extremist beliefs generally accept the possibility of a violent solution, which is normalized and justified as a means to an end (23, 24). Given the disruption that such groups inflict on society, there is a strong motivation to understand their behavior, including from the point of view of their psychological motivation and mental health. The literature has largely argued that those who engage in terrorism are psychologically stable and lack any enrichment for particular personality traits or specific mental disorders (25), although in individual cases the evidence can be substantial. On the other hand, research that has focused specifically on *lone-wolf terrorism* has identified a significant increase in depression, anxiety and specific personality disorders (26). In the context of the current article, many are loners who have very few if any social relationships, and some identify with or have been formally diagnosed with ASD. Of course, the episodic nature of disorders such as depression and schizophrenia means that the risk may come and go, as symptoms deteriorate and improve. The fluctuation in risk associated with these disorders will also relate to contact with services for support and monitoring.

One notable study examined the prevalence of specific mental health disorders among group (*N* = 158) and lone-actor (*N* = 119) terrorists using retrospective data comprising reports from a variety of sources (26). Cases were not directly evaluated and so at best the “diagnoses” are speculative. Nonetheless, 3% of the lone actor terrorists were labeled ASD, compared to the population prevalence of 1.5%. One illustrative example is Sandy Hook in Connecticut where a 20-year-old murdered 26 people by shooting, among whom were 20 children. At the age of 13 years the perpetrator was diagnosed with Asperger Syndrome (now recognized under the umbrella term ASD) and a year later also with obsessive compulsive disorder (OCD). He was described as socially aloof, awkward and emotionally disengaged even from family. He developed political interests and opinions that he argued with authority and arrogance (27). His OCD manifested in a typical repetitive and ritualistic manner, driven by sensory overload and fear of contamination, as was his anxiety and panic attacks. Even given this small amount of clinical information it would be very difficult to argue against the relevance of his diagnoses, which has been discussed in detail elsewhere (27).

In some cases, diagnosis is made post offense, often in the context of Court-directed psychiatric and psychological assessment. Even now it is not unusual for diagnosis of ASD to be delayed into adulthood, and so this is not particularly indicative of their symptoms being relatively “milder” than those diagnosed when younger. As many extremists who carry out terrorist activities ultimately also kill themselves, or are killed by law enforcement, diagnosis is also sometimes based on an indirect means (9, 10), such as interview with family, friends and documentation from diaries or blogs rather than through direct assessment. These “diagnoses” must be approached with a degree of skepticism.

## Relevant Risk Factors

Vulnerability to extremist patterns of thinking may be related to ASD's rigidity and otherwise ritualistic and constrained patterns of belief and behavior. The manifestation may vary from individual to individual, for some manifesting as circumscribed interests that are pursued with intensity. The focus of interests vary, but information gathering on particular topics is frequent. It is recognized that there may be an association between circumscribed interests and violence (28–30), although the focus of interests themselves is not a good marker of risk: for example, a fixation on military or law enforcement may be confined purely to an academic interest.

For some, patterns of thinking may be rigid, with fixed, firmly held beliefs. This may be an extension of a particular interest, but may also relate to a personal experience. Although people more generally may become emotionally involved with particular topics, for an autistic person this may become all encompassing, and, importantly, held without consideration of counterarguments. In clinical practice, such rigidly held beliefs are not uncommon, and for the most part result in further alienation, compounding any depression or anxiety, but not resulting in violence. For others, however, these beliefs can be acted out in the form of violence (31).

What may be more important than particular interests or rigidly held beliefs is the social exclusion and bullying that are both common among adolescents with ASD, and which can fuel resentment and hate toward their peers. The adolescent years place particular emphasis on social performance as a metric for “worthiness,” and can be brutal for someone who struggles to understand interpersonal behavior. Numerous first-person narrative accounts of individuals with ASD describe the sense of hopelessness imbued by the observation that the most socially competent individuals are always the most popular, not only among peers but also teachers [for example (32)]. Children and adolescents with ASD are often the victims of bullying (33), sometimes over a long period of time. This may result in feelings of anger toward others, particularly victimizers and their affiliates, even among those *without* ASD but who have engaged in mass violence. The exact relationship between bullying and mass violence is unclear however: some evidence suggests that 87% of school shooters had described of being victims of bulling (34), whereas a more detailed examination of school shooters found no such evidence (35).

On the one hand, therefore, extremism-mediated violence among those with ASD may not be so much about ASD *per se*, but instead relate to how others treat them because of their vulnerabilities. The appeal of belonging, such as by associating with an outgroup, may be stronger in people who do not experience a sense of community and belonging in other contexts. Of course, other factors that have not been well-studied, such as poor empathy or mentalizing, may also mediate violence through their impact on mechanisms that inhibit violence (13, 36). Research has also shown that a history of trauma can impact the same biological mechanisms and may in itself be associated with an increased risk of violence (37). However, trauma as a precursor to poor outcomes among people with ASD has not been well-studied, and hence the role of childhood sexual and physical abuse and neglect on the risk of violence specifically in ASD is unknown (38).

## Information Seeking and Anxiety Reduction

Individuals with ASD are at higher risk for anxiety disorders and difficulties (39–41). This is also a population where familiarity with computers and the internet is common and indeed frequently seen as a life-line with great fluency with web based resources (42, 43). This fluency has both positive and negative effects (44). For example, there is potential for anxiety reduction through information gathering, but, conversely, internet searching can become obsessive and the potential for internet addiction is high (45, 46). One of the great risks is that lack of good social judgment and supportive peers (who can provide an important balance to disinformation) makes individuals with ASD more vulnerable as targets for misinformation (47).

Instances of terrorism may lead individuals with Asperger's or ASD who are cyberfluent to seek information initially to manage their anxiety. However, repeated internet searches on topics related to terrorism (e.g., building bombs or information on terrorist groups) may be noticed by government agencies even if the individual involved is merely curious. On the other hand, sometimes the lack of social sophistication, critical thinking, and poor judgment can act to seemingly or actually radicalize individuals with ASD (48). It is important that supporters and family members help provide good information on safe internet use and that government agencies be aware of these potential vulnerabilities.

## Assessment

There is much to be gained from developing a better understanding of the relative roles of these various factors in the etiology of violence among individuals with ASD, crucially to inform prevention and treatment. Assessment of individual motivation is often performed by suitably trained professionals as expert witnesses in order to inform decisions of culpability. In this way, the whole gamut of possibly relevant etiological factors can be considered along with other stochastic events and decisions the defendant may have made along the way. The expert who considers individual level risk has the opportunity to pull together these multiple threads into a rich, detailed understanding that surpasses the generic, descriptive actuarial risk. It is not enough to say that ASD is or is not relevant; reducing risk in this simplistic way will for certain undermine the ability to understand the nature of risk and how best to manage that risk.

The actual assessment itself requires the use of standardized instruments both in the diagnostic assessment itself but also in relation to measuring additional relevant traits and behavior such as acquiescence/suggestibility, mental health-comorbidity, and theory of mind/empathy. Assessments are widely available, a discussion of which is out of the scope of this current article. However, it is important to bear in mind that none of the ASD diagnostic assessments were developed to be used in a forensic population, and may not be sensitive to differentiate between, for example, social impairments that are largely trauma related from those that may be indicative of ASD. Given that adult assessments place a greater emphasis on adult adjustment rather than development, this may be particularly problematic. Similarly, screening assessments were developed for community use, and the questions posed are biased toward particular experiences that may not reflect those who have had less opportunities.

One potential drawback of focusing on diagnosis is that in the search for mental health “explanations” the true complexity of human behavior may be missed. As indicated above, there is a need to understand extremism and extremist-related behavior, but it is often the case that in doing so explanations offered become pallid and rather simplistic. Moreover, by simply looking for diagnostic labels, there is a chance that such reductionist tendencies merely identify “red herrings” rather than true risk factors. A related issue is the tendency to apportion “responsibility” for behavior to a particular diagnostic label, when in fact the role it plays maybe really quite small and peripheral. One reason this may happen is that experts are trained to focus on specific dimensions of risk, whether psychiatric, psychological, social, environmental, or economic. Indeed, understanding extremism and terrorism/mass violence has drawn focus from different academic disciplines as well as legislators, policy makers and security experts. What is striking is the broad-based expertise that characterize those working in this area. Although this carries the option of a fruitful exchange of information, more often than not true cross-fertilization of knowledge is confounded by little opportunity to work together, or other agendas, political, or otherwise.

## Discussion

The motivation for an individual to hold extremist beliefs and the risk of violence represent the interplay between a number of factors, both well-defined and those that are stochastic and unpredictable. We have considered risk from a mental health perspective, and specifically in relation to ASD. People with ASD are certainly represented among those who are affiliated with extremist groups, and some have engaged in extremist-related violent acts. Whether this is more frequent than those with other neurodevelopmental disorders, or those with other mental health problems, is unclear. We expect that the relationship is not strong, but at the same time, as articulated above, there are reasons to believe that the diagnosis will be relevant. In order to be able to approach these difficult clinical questions, what is needed is suitably large-scale research that draws on expertise across disciplines and that goes beyond the level of diagnosis to explore trait and neuropsychological risk factors, both current and historical. Although case studies are informative, they do not allow hypotheses concerning, for example, risk and protective factors to be tested. Moreover, given the multitude of mental disorders raised in the extant literature, and the strong possibility of individuals displaying co-morbidity, there needs to be careful attention to characterizing the clinical phenotype. Good clinical practice dictates that at every stage of the criminal justice process ASD and other neurodevelopmental diagnoses are given appropriate consideration when formulating cases with reference to culpability, criminal intent and other aspects of criminal responsibility.

## Data Availability Statement

The original contributions presented in the study are included in the article/supplementary material, further inquiries can be directed to the corresponding author/s.

## Ethics Statement

Written informed consent was not obtained from the individual(s) for the publication of any potentially identifiable images or data included in this article.

## Author Contributions

MW-S wrote the first draft and all authors subsequently contributed to revisions. All authors contributed to the original conception of the idea, contributed to the article, and approved the submitted version.

## Conflict of Interest

The authors declare that the research was conducted in the absence of any commercial or financial relationships that could be construed as a potential conflict of interest.

## Publisher's Note

All claims expressed in this article are solely those of the authors and do not necessarily represent those of their affiliated organizations, or those of the publisher, the editors and the reviewers. Any product that may be evaluated in this article, or claim that may be made by its manufacturer, is not guaranteed or endorsed by the publisher.
